# Fluorescence Adherence Inhibition Assay: A Novel Functional Assessment of Blocking Virus Attachment by Vaccine-Induced Antibodies

**DOI:** 10.1371/journal.pone.0144261

**Published:** 2016-02-10

**Authors:** Atul Asati, Olga Kachurina, Alex Karol, Vipra Dhir, Michael Nguyen, Robert Parkhill, Diana Kouiavskaia, Konstantin Chumakov, William Warren, Anatoly Kachurin

**Affiliations:** 1 Sanofi Pasteur VaxDesign Campus, 2501 Discovery Dr. Suite 3000, Orlando, Florida 32826, United States of America; 2 U. S. Food and Drug Administration, Center for Biologics Evaluation and Research, 10903 New Hampshire Avenue, Silver Spring, MD 20993, United States of America; Icahn School of Medicine at Mount Sinai, UNITED STATES

## Abstract

Neutralizing antibodies induced by vaccination or natural infection play a critically important role in protection against the viral diseases. In general, neutralization of the viral infection occurs via two major pathways: pre- and post-attachment modes, the first being the most important for such infections as influenza and polio, the latter being significant for filoviruses. Neutralizing capacity of antibodies is typically evaluated by virus neutralization assays that assess reduction of viral infectivity to the target cells in the presence of functional antibodies. Plaque reduction neutralization test, microneutralization and immunofluorescent assays are often used as gold standard virus neutralization assays. However, these methods are associated with several important prerequisites such as use of live virus requiring safety precautions, tedious evaluation procedure and long assessment time. Hence, there is a need for a robust, inexpensive high throughput functional assay that can be performed rapidly using inactivated virus, without extensive safety precautions. Herein, we report a novel high throughput Fluorescence Adherence Inhibition assay (fADI) using inactivated virus labeled with fluorescent secondary antibodies virus and Vero cells or erythrocytes as targets. It requires only few hours to assess pre-attachment neutralizing capacity of donor sera. fADI assay was tested successfully on donors immunized with polio, yellow fever and influenza vaccines. To further simplify and improve the throughput of the assay, we have developed a mathematical approach for calculating the 50% titers from a single sample dilution, without the need to analyze multi-point titration curves. Assessment of pre- and post-vaccination human sera from subjects immunized with IPOL^®^, YF-VAX^®^ and 2013–2014 Fluzone^®^ vaccines demonstrated high efficiency of the assay. The results correlated very well with microneutralization assay performed independently by the FDA Center of Biologics Evaluation and Research, with plaque reduction neutralization test performed by Focus Diagnostics, and with hemaglutination inhibition assay performed in-house at Sanofi Pasteur. Taken together, fADI assay appears to be a useful high throughput functional immunoassay for assessment of antibody-related neutralization of the viral infections for which pre-attachment neutralization pathway is predominant, such as polio, influenza, yellow fever and dengue.

## Introduction

**Fig 1:**
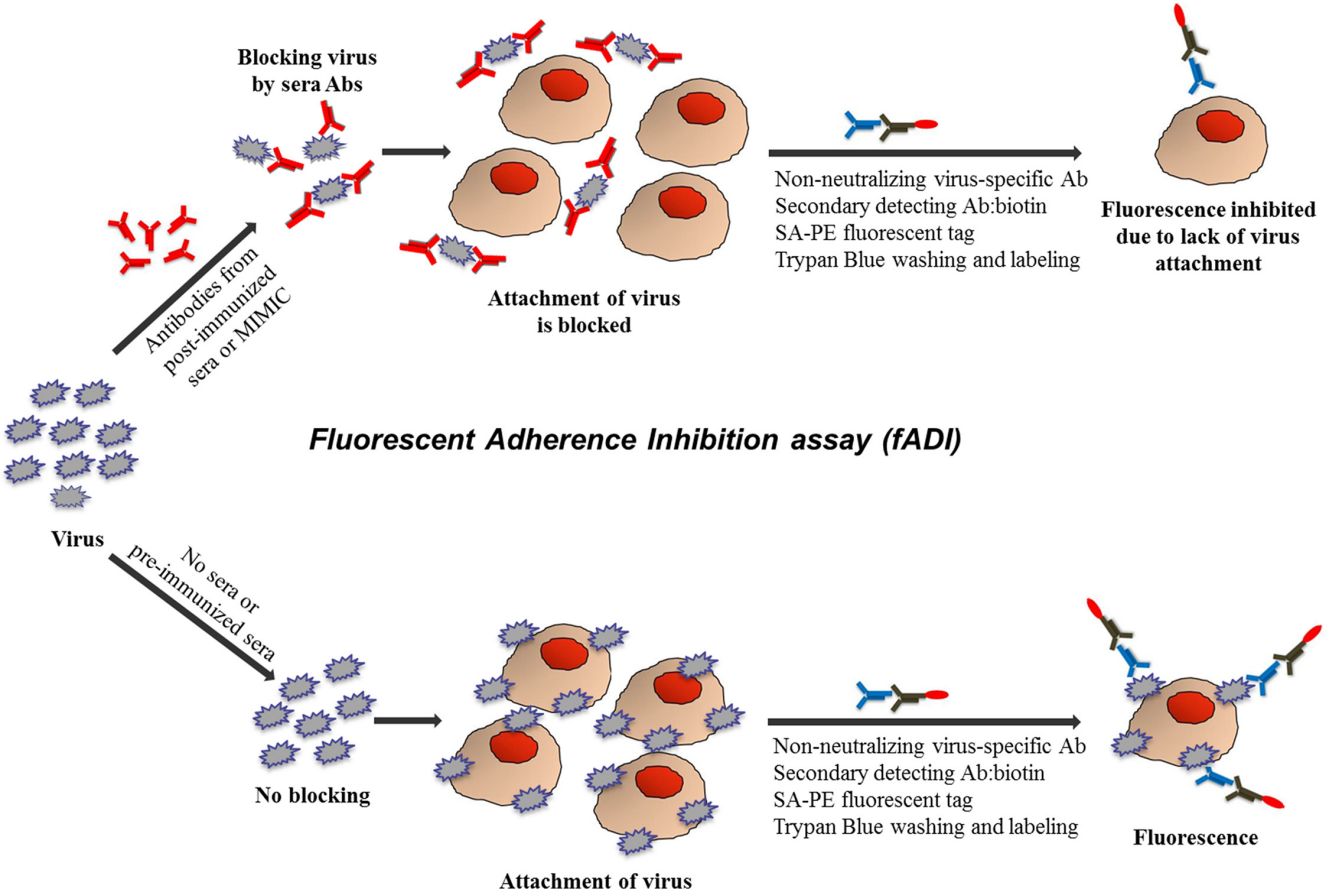
Schematic representation of fADI neutralization assay. Incubation of virus with sera (upper arm) or without sera (lower arm) either blocks or does not block virus attachment to target cells, respectively. Virus attached to the cells is detected by virus specific antibody and fluorescent tag SA-PE followed by additional staining cells with diluted Trypan blue and finally reading on Bioplex. The maximum fluorescent signal without serum corresponds to zero blocking, (lower arm), reduced fluorescent signal indicating blocking of the virus with serum antibody (upper arm).

**Fig 2:**
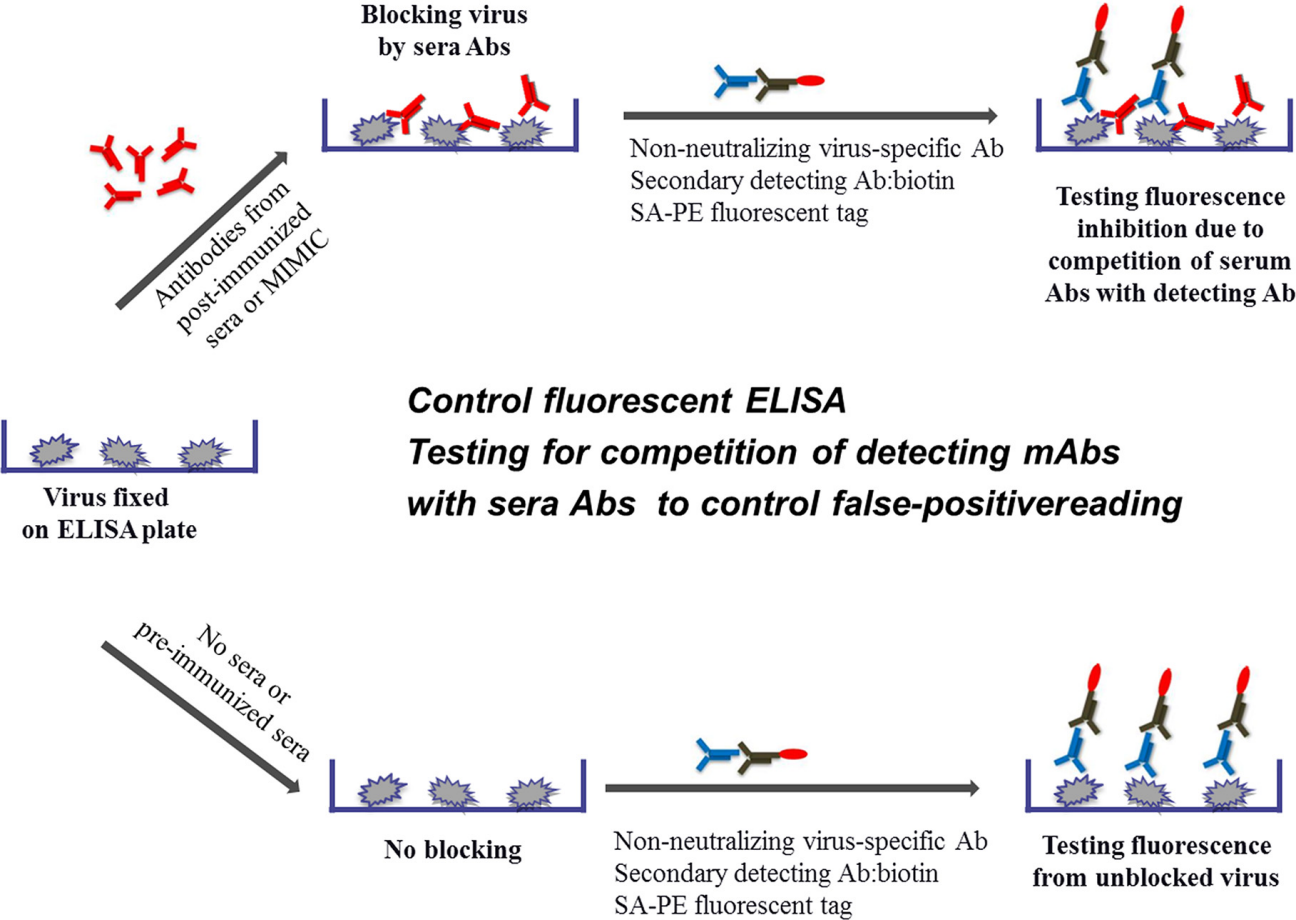
Schematic representation of Control ELISA to check for false positive effects in the fADI neutralization assay. Virus is added to ELISA plates instead of using target cells to check for any false positive readings. Addition of sera (upper arm) or no sera (lower arm) should produce similar reporter signals (fluorescence) indicating the absence or low level of *competition* of virus-specific labeling antibody with antibodies in test serum. The plates are read on the Synergy HT plate reader (BioTek) after applying the secondary detecting antibody and the fluorescent tag SA-PE. More details are in the text.

Antibodies that able to neutralize viral infections can work by two major pathways of actions: pre- and post-attachment mechanisms. The latter can include blocking of endosomal processing of the virus, subsequent membrane fusion and release of the capsid content or, alternatively, blocking the release of virus progeny from the host cells. Post-attachment pathway is known as very important for neutralizing filoviruses. On the other hands, pre-attachment blocking remains the most important neutralizing pathway for influenza, polio, and dengue, and for those infections the ability of antibodies to block attachment of the virus to the target cell remains the most important property of the immune response to viral pathogens. Among various functional methods to assess antibody neutralizing capacity, plaque reduction neutralization test (PRNT), ELISA-based endpoint assessment microneutralization assay (MN) and indirect immunofluorescence assay (IFA) are widely used to determine the functionality of serological response to vaccinations. 50% neutralization titers of sera obtained in these assays are widely accepted as correlates of protections [1–15]. However, these methods are associated with several limitations such as the need to use live virus, requiring safety precautions, tedious, expensive procedure and long assessment time. According to the World Health Organization (WHO), after polio eradication poliovirus will require Biosafety Level III (BSL III). Similarly, dengue virus has been classified as BSL III agent in European countries [16]. All these factors further increase the need for using methods based on inactivated viruses. Advances in flow cytometry technique promises to significantly improve existing neutralization assays [17–25]. Although assays based on flow cytometry are sensitive and informative, their use is limited because they require live viruses or recombinant vectors, which raises safety concerns and requires sophisticated laboratory setups. Here we describe a fast, robust, and sensitive technique for assessment of specifically pre-attachment neutralization that utilizes inactivated virus labeled with fluorescent secondary antibodies, allowing tests to be performed in 96-wells format without extensive safety precautions and expensive laboratory equipment. We named the method as fluorescent Adherence Inhibition assay (fADI) [26]. The principle of fADI is depicted in Fig 1. The virus is incubated with the test sera samples from vaccinated subjects (the upper part of the scheme in Fig 1), or with a buffer or medium containing no sera (the lower part of the scheme Fig 1), followed by addition of the target Vero cells. Next, a virus-specific antibody from non-human species is added followed by biotinylated species-specific secondary antibody and fluorescent streptavidin-phycoerythrin tag (SA-PE). Cells are read on Bioplex-100 used as a micro-FACS device. Since fADI assesses pre-attachment neutralization pathway, i.e. blocking of attachment of the virus to the target cells (the very first stage of virus infection), the whole experiment is performed at 4°C which prevents internalization of the virus by endocytosis [27–28]. Virus attachment to Vero cells is expected to be blocked in samples that contain neutralizing antibodies. This will be manifested in the decrease of the fluorescent signal (the upper part of the scheme in Fig 1), while no-serum control sample will exhibit high fluorescent signal indicating maximum virus attachment on the cells (the lower part of the scheme in Fig 1). The ratio of the fluorescent signals obtained in the upper and the lower pathways shown on Fig 1 gives Virus Attachment Index (VAI). VAI closer to 1 indicates no blocking activity, while VAI close to 0 means complete blocking. Additionally, in order to control for the possible false results due to competition between the virus-detecting antibody and antibodies in the tested sera, an additional ELISA assay is performed at the conditions similar to those in the main fADI experiment, except that the virus is attached to the surface of the ELISA plates instead of the target cells (Fig 2). If the VAI values calculated for the control ELISA are considerably less than 1, it indicates competition between the detecting and sera antibodies. However, for properly selected detecting antibodies and optimized experimental conditions, majority of control ELISA tests showed low or no competition, and consequently no significant false blocking effects were observed in the fADI experiments. As a result, control ELISA may be considered not a mandatory part of the fADI assay, once no significant competition of the detecting antibodies and sera antibodies are demonstrated in a given study.

## Results

### fADI Proof of Principle

**Fig 3:**
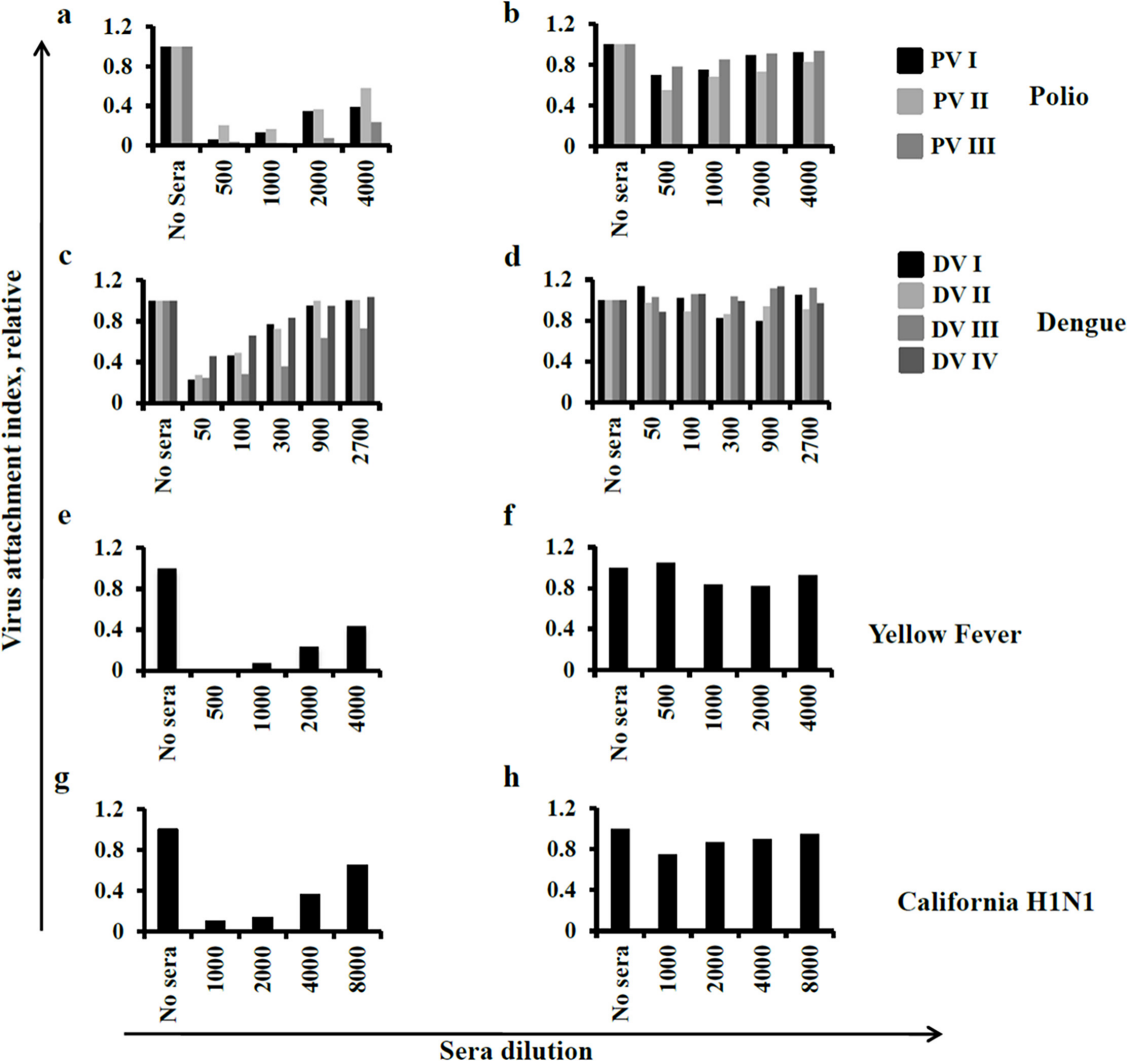
Proof of principle study for the fADI assay against different classes of viruses. fADI displayed a significant sera concentration dependent blocking of virus attachment for polioviruses of serotyopes I, II and III (A), Dengue I, II, III and IV (C), Yellow Fever (E) and influenza California H1N1viruses (G) to Vero cells. The sera used in the demo experiments were standard hyper-immune sera specific to the corresponding viruses. Control ELISA (B, D, F and H) tests with those standard sera did not display any significant reduction in fluorescent signal thus showing very low or no competition between the sera antibodies and virus specific labeling antibodies used in the assay.

In the preliminary sets of experiments, we used a variety of human and animal standard anti-sera against different viruses such as poliovirus of three serotypes (PV I, II, III), dengue viruses of four serotypes (DV I, II, III and IV), Yellow Fever virus (YF) and Influenza A virus (California H1N1). This was done was to demonstrate the utility of fADI assay to detect antibodies to a wide range of viruses. Additionally, we carried out comparative fADI experiments using Marburg and Ebola viruses using convalescent non-human primate sera, and Brisbane H1N1 influenza virus using pre- and post-immunized human sera. The result of this comparative study represented an explicit confirmation of the prevalence of post-attachment endosomal neutralization pathway for filoviruses [29]. and, conversely, pre-attachment neutralization pathway for influenza (S1 Fig). All proof-of-concept fADI experiments were carried out in 96-well formats at 4°C using various dilutions of standard sera, depending on the type of the virus used in the assay. In all cases, dilution-dependent inhibition of virus attachment was observed (Fig 3A, 3C, 3E and 3G). At the same time, ELISA control tests carried out using experimental conditions identical to those used in the main fADI experiments did not show significant reduction of fluorescence intensity (no false blocking effect) caused by competition between the detecting antibodies and sera antibodies (Fig 3B, 3D, 3F and 3H). These results indicated that fADI assay does exhibit blocking of virus attachment in presence of functional antibodies in virus-specific standard sera.

### fADI assay using turkey erythrocytes for Influenza viruses

Next, we compared fADI assay for influenza A viruses with turkey erythrocytes as targets with the classical HAI assay using human erythrocytes (type “O” group). A total of 36 pre- and post-immunized sera obtained from donors vaccinated with 2006–2007 influenza vaccine were tested. The results showed that fADI worked efficiently with turkey erythrocytes and demonstrated strong positive correlation with HAI for both New Caledonia H1N1 and Wisconsin H3N2 viruses (S2A and S2B Fig). These preliminary results showed considerable flexibility of the fADI method, comparability with traditional assays and the ability to efficiently discriminate between immune responders and non-responders.

### fADI assessment of IPV^®^ vaccinated human clinical serum and formulation of mathematical model to determine 50% blocking from single sample dilution

**Fig 4:**
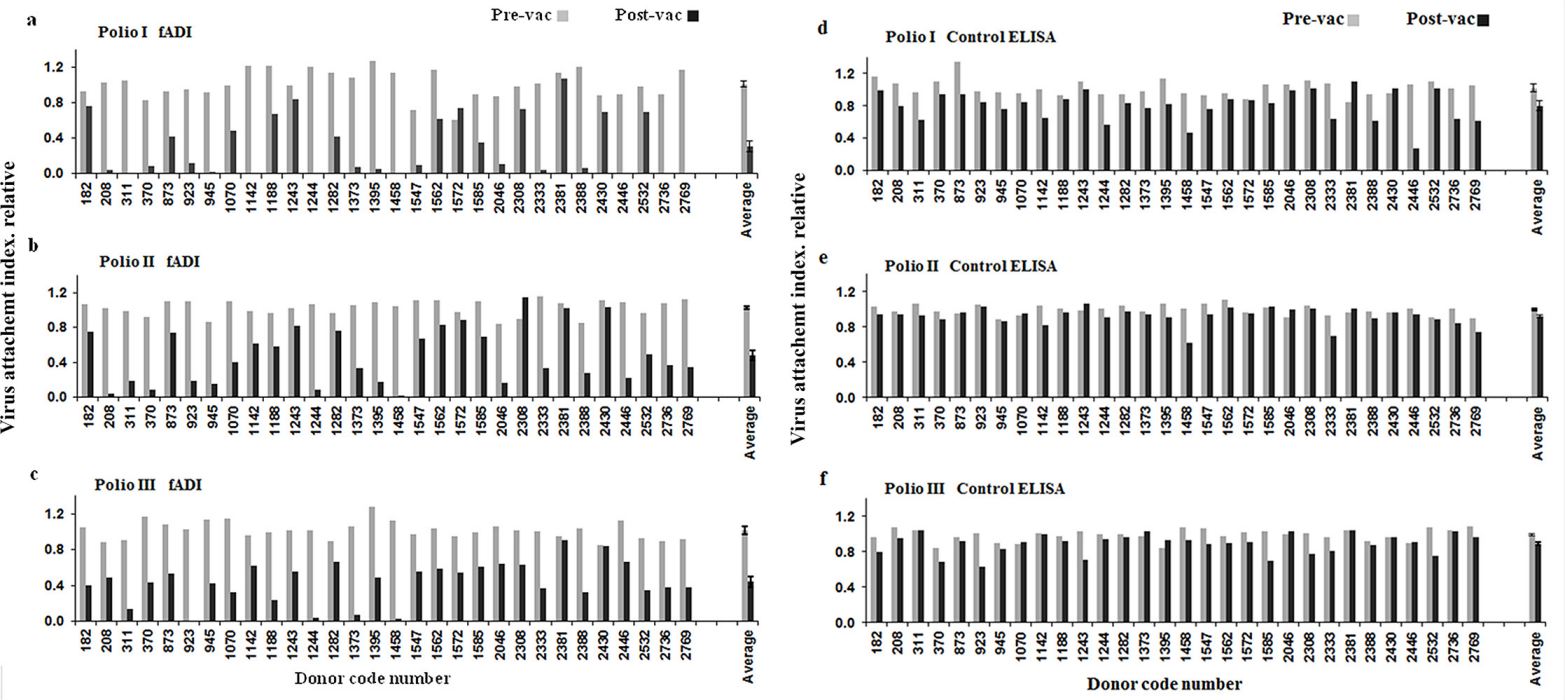
fADI screening of pre- and post-vaccinated serum samples from donors immunized with IPOL^®^ vaccine. The screening was performed in the single dilution mode rather than using sera titration. Donor-to-donor variation in the capacity of donor sera to block virus attachment was observed for all three poliovirus serotypes (A, B and C). The control ELISA tests (D, E and F) did not display significant reduction of the reporter fluorescence signal, thus showing very low or zero competition between test serum antibodies and virus-specific labeling antibodies used in the assay.

Next, fADI assay was used to evaluate the antibody response in human clinical sera obtained from 30 human donors immunized with inactivated poliovirus vaccine (IPV). For that purpose, both pre- and post-vaccination human sera were tested in the fADI assay against formaldehyde inactivated Polio I, II and III viruses (Sanofi Pasteur). Since the rationale behind developing fADI was to develop an express and high throughput functional immunoassay, blocking capacity of the entire cohort of donor sera was measured at a single dilution of 1:1000 (Fig 4A–4C). fADI results showed considerable donor-to-donor variations in the capacity of their post-vaccination sera to block attachment of polioviruses to Vero cells, clearly distinguishing them into high-, mid- and low-level responders, as opposed to very insignificant blocking activity for most of the pre-immunized sera. Control ELISA carried out under experimental conditions identical to those used in the main fADI experiment revealed a certain level of competition between the detecting antibodies and the antibodies for a small number of sera for Polio I assay, and no significant competition for Polio II and Polio III assays, thus indicating that the false blocking effects are minimal (Fig 4D–4F). Nevertheless, it was found useful to mathematically compensate the fADI data for even minor residual competition effects. For that matter, the final fADI VAI number was normalized by the VAI numbers calculated from the control ELISA data:

fADIVAIcorr=(fADIVAImeasured)/(ELISAVAI). (1)

The normalization (1) compensates for small false-blocking effects in some samples. Altogether, the fADI VAI dataset obtained from IPOL-vaccinated donors demonstrated a potential of the fADI to be used for express evaluation of the clinical results of after immunization with IPV.As was already mentioned, the rationale behind development of fADI was to create a functional immunoassay for express high throughput measurement of virus-blocking activity of human sera. Most of fADI experiments were performed using a single dilution of the sera samples. This approach simplifies comparison of the blocking activity of a large number of samples in a single test. As explained previously, VAI determined in fADI measurements and defined as the ratio of fluorescent signal obtained from sera samples to no sera samples. VAI numbers are informative and explicit. However, immunologists and virologists typically use multiple point titration techniques for various neutralization methods that involve serial dilutions of sera samples and determine sera efficiency by calculating the 50% titers of neutralization. To facilitate comparison of the express fADI results with data obtained by other methods, we have used a mathematical calculation that allowed conversion the VAI data obtained at single dilution of sera samples, into the 50% blocking titers. VAI numbers and blocking titers of the test sera correlated very well. VAI values close to 1 represent low neutralizing titer in the test sera, while the VAI values close to zero correspond to high neutralizing titers, even though the relation between the VAI numbers and the neutralizing titers is not linear. Immunizations often produce immune responses that vary between individuals, therefore the sera samples contain different levels of blocking antibodies, and the titration curves may also be different. However, assuming that the *shapes* of the titration curves for different sera samples are similar, extrapolation could be used to derive the point of 50% titration (VAI = 0.5) from just one arbitrary titration data point where the VAI was determined. This can be done by using the equation that describes the *general shape* of titration curve, as it is shown schematically on S3 Fig. In this scheme, the VAI numbers taken from fADI titrations of two randomly selected sera samples, A and B are transferred on the symmetrized titration curve that reproduces the *general shape* of sera titrations. The symmetrized approximating curve allows calculation of how much a given serum dilution should be decreased (for Serum A) or increased (for Serum B) to reach the point of titration where VAI = 0.5. Next, to find the formula that would link the VAI data to the sera titers, we applied a method of sequential approximations, where a rough relation between the two categories is set as the initial approximation using a limited volume of data, and then improved in a few subsequent steps using additional data, as it is shown in S4 Fig. In the beginning of the approximation procedure, we used fADI titration of an arbitrarily chosen donor serum, starting at a dilution factor of 125. At the end of approximation, when it became obvious that the titration curves for different sera have similar shapes, the resulting approximating curve was symmetrized as it was shown in S3 Fig. To better approximate sera titrations, we tried a few formulas for S-shape curves, out of which the simplest one gave the best fitting:

Y=1/(1+Exp(a−b*Ln(X))) (2)

*a* and *b* are digital parameters to be determined using Least Square Fitting procedure, X and Y are the Relative Sera Dilution factor and VAI number, respectively. After refinement via the Least Square Fitting procedure and symmetrization, the formula connecting fADI titers and VAI values has transformed into the following final form:

fADI50%titer=(Serumdilutionfactor)*Exp((0.44428−(1.495−Ln((1−VAI)/VAI))/3.365)*Ln(10)) (3)

This formula allowed us to calculate fADI 50% titers from the single dilution VAI data. S5 Fig illustrates the results of such calculations for polio fADI. Conversion of the VAI data to 50% titers works satisfactorily in the VAI range of ~ 0.05–0.90. It provides only rough estimations of the titers, of course, but can serve as a convenient instrument for express assessment of sera samples in familiar units when experimental time is limited. S5 Fig shows good reproducibility of the calculated fADI 50% titers for a wide range of the primary VAI data.

### Comparison of Polio fADI versus MN performed at CBER, FDA

**Fig 5:**
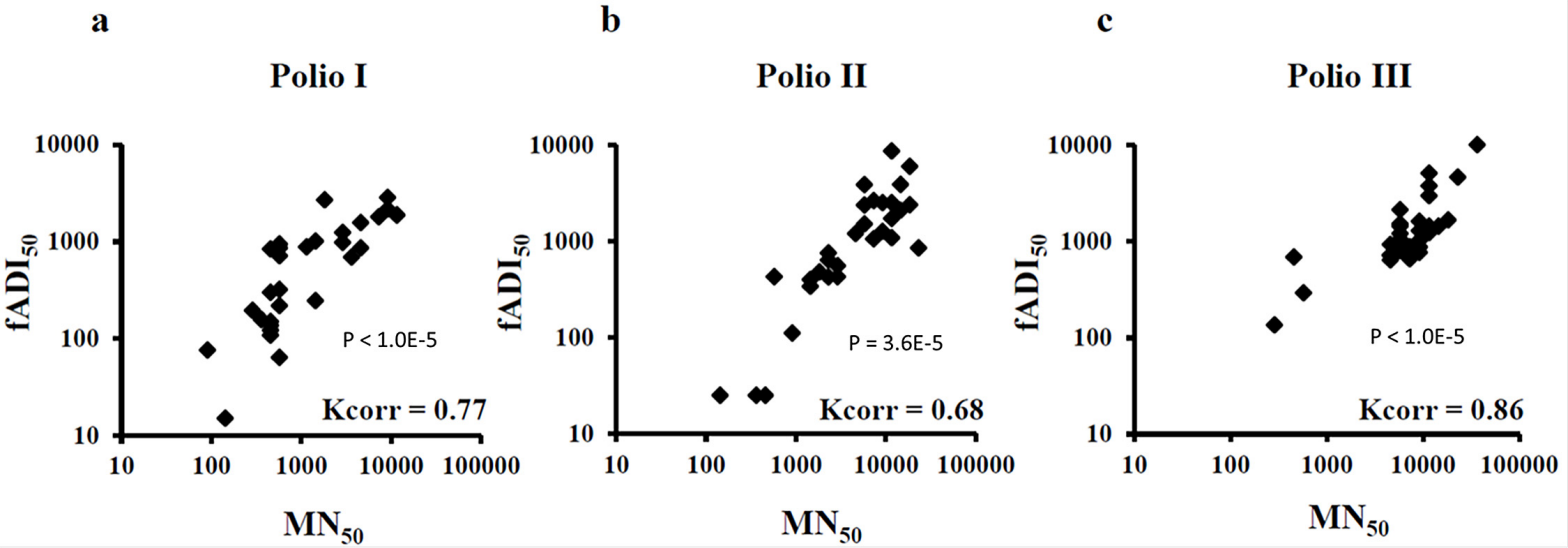
Correlation between fADI and MN methods for Polio I, II and III. fADI_50_ were calculated from the Virus Attachment Indexes using a mathematical approximation model (see text for details). fADI showed a significant correlation of virus attachment blocking for polioviruses of serotypes I, II and III with the data of microneutralization assay obtained independently at CBER/FDA using the WHO miconeutralization protocol.

To evaluate the validity and feasibility of a new functional immunoassay, it is essential to compare it with the other established standard techniques, therefore we compared fADI data obtained for sera poliovirus-immunized individuals with the results of the classical and conventional microneutralization assay (MN). The same donor sera that were screened using fADI assay were evaluated at the Center of Biologics Evaluation and Research (CBER) laboratory of Food and Drug Administration (FDA) using MN protocol recommended by the World Health Organization (WHO) [4]. Since the data of the MN assay are normally expressed in 50% titers, we calculated the corresponding fADI 50% titers from the original VAI format using formula (3) described above (S6 Fig). The MN and fADI titers plotted in the dual scattered manner showed considerable correlation between the two methods with p value around of, or lower than 1.0E-5 (Fig 5A–5C), which further supports possibility to calculate 50% titers from one-point VAI data in express sear screening.

### fADI assessment of YF-VAX^®^ vaccinated human clinical sera and comparison with PRNT Golden Standard assay

**Fig 6:**
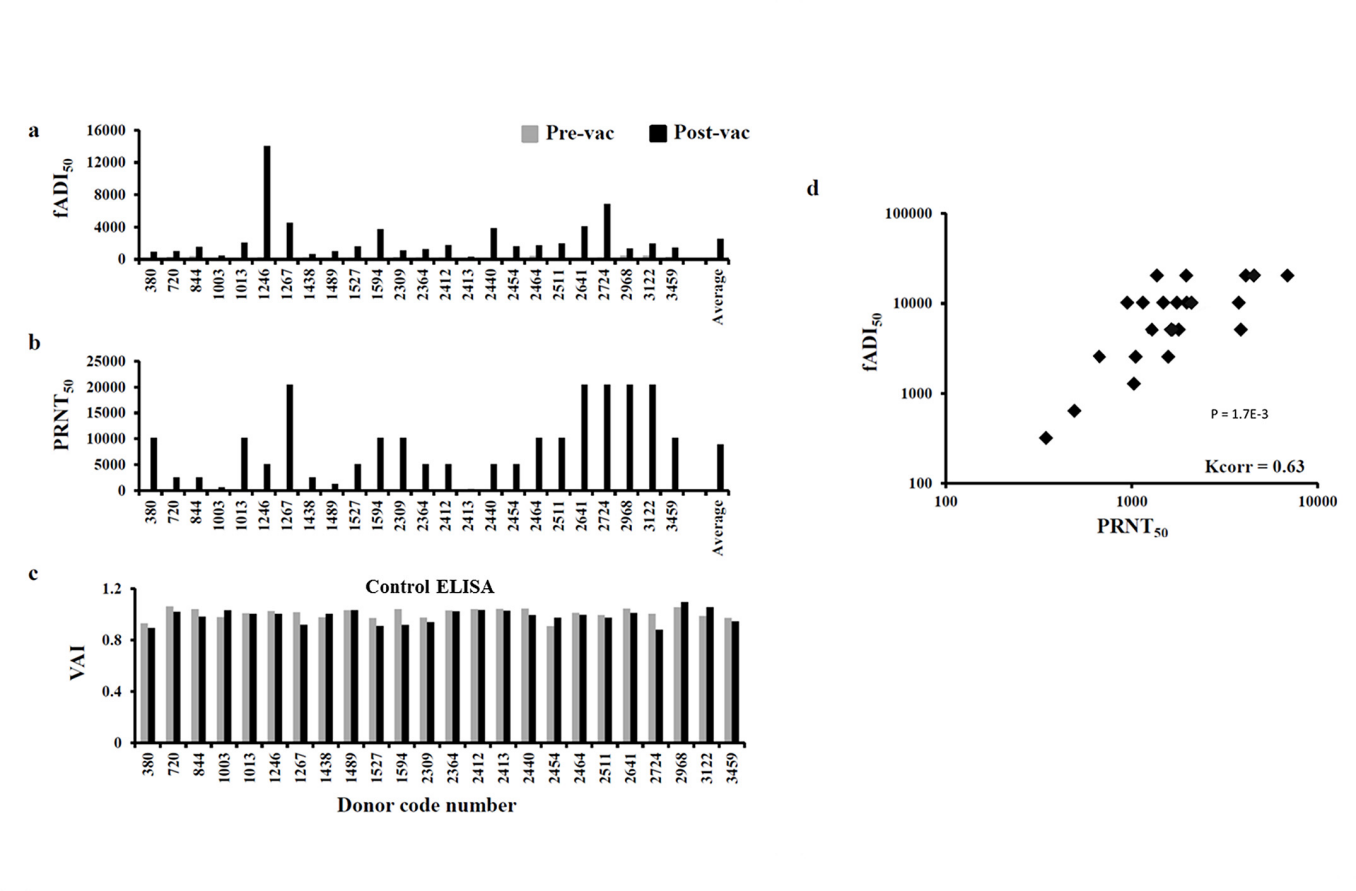
Correlation between fADI and PRNT methods for Yellow Fever vaccine virus. fADI_50_ were calculated from the Virus Attachment Indexes using a mathematical approximation model, more details in the text. The fADI titers for all the pre-vaccination sera samples were found very low. fADI displayed a considerable donor-to-donor variation in the capacity of post-vaccination donor sera to block attachment of vaccine strain 17DD of Yellow Fever virus to Vero cells (a). PRNT_50_ data were obtained for the same sera samples independently by a commercial vendor (b). Control ELISA did not reveal a reduction of the reporter fluorescence signal, thus showing zero competition between the sera antibodies and the virus specific labeling antibodies used in the assay (c). A considerable positive correlation between the fADI and PRNT data for post-vaccination sera samples was found (d).

Similarly, we applied fADI for assessing attachment-blocking effect of human sera from individuals immunized with Yellow Fever vaccine YF-VAX^®^. For assessing YF vaccinated sera, we used UV-inactivated Yellow Fever vaccine 17D virus (Sanofi Pasteur). Both pre- and post-immunized sera samples were tested at dilution factor of 500. Concurrent ELISA test was performed to control for a possible false positive blocking effect, as described above. The results revealed a considerable blocking of virus attachment to Vero cells for sera immunized with Yellow Fever (Fig 6A, 6B and 6D), while control ELISA tests did not show any significant decrease in fluorescent signal, thus indicating to no false blocking (Fig 6C). Classically, PRNT has been used as a gold standard neutralization assay for determining neutralizing antibody 50% titer and shown to be the correlates of protection against yellow fever virus infection [14–15]. Hence, to determine the validity of fADI, PRNT was carried for the same pre- and post-immunized donor sera independently in the laboratory of Focus Diagnostics Inc., California, USA. We found a significant positive correlation between fADI and PRNT for Yellow Fever (Fig 6D).

### Automation of fADI assay

Converting cell based assays workflow from manual to automated mode typically enhances the analytical capacity, throughput, robustness and gives scientists freedom to perform more value-added tasks. Additionally, it decreases operator-dependent variations making assay more consistent and reproducible. Therefore, we decided to automate fADI to further improve the efficiency and acceptability of assay. To prove this, we have performed both manual and automated fADI assays simultaneously for randomly selected 20 sera collected from individuals before and after immunization with IPOL^®^ 2009 poliovirus vaccine. This set of samples was different from the one used in the earlier study shown on Fig 4. The automated assay was performed using Beckman Coulter Biomek^®^ workstation with SAMI^®^ EX scheduling software and custom scripting through Biomek^®^ software. The data was converted from VAI to 50% titers using mathematical model discussed in the previous sections. There was a two-fold increase in fluorescence signal intensity in the automated mode, which allowed us to use lower concentration of virus and higher sample dilutions, thereby increasing the overall sensitivity of fADI assay. In addition, a strong correlation between manual and automated modes of fADI was observed for all three serotypes of poliovirus, in both VAI and 50% titer scales (S7A–S7E Fig). Therefore, we proved that fADI is a robust surrogate for the neutralization assay with a simple protocol that can be easily automated for high throughput screening and increased assay efficiency, suitable for rapid screening of a large number of clinical samples.

## Discussion

In conclusion, an advanced express functional immunoassay was developed for assessment of vaccination-induced antibodies responsible for blocking virus attachment to the target cells, which can be done within few hours using inactivated virus. fADI as a functional assay that exhibited high efficiency in determining blocking of the virus attachment, thus measuring the very first stage of viral infection and complementing other assays for various neutralization mechanisms. Moreover, fADI demonstrated considerable flexibility allowing massive sera screening in a single dilution mode or multiple dilution titration mode as needed. The approach uses a mathematical formula to calculate 50% titers from a single-point VAI data. The fADI assay has demonstrated strong correlation with existing gold standard assays. Automation of the fADI assay makes it even more suitable for high throughput screening. Therefore, the proposed fADI assay could be used as a surrogate for neutralization tests to study vaccine-induced antibodies in human sera in large clinical trials of human sera.

## Materials and Methods

### Donor blood products

Blood products were obtained from One Blood (Orlando, FL). This study was reviewed and approved by Chesapeake Research Review Inc. (Columbia, MD). All donors had signed a written informed consented and found to be in good health at the time of collection. All blood products were tested and found negative for bloodborne pathogens as detected by standard blood bank clinical assays

### Miscellaneous materials

Standards sera, viruses and assay reagents were obtained from the following sources. Rabbit anti-polio serum (I, II and III) was provided by Center of Biologics Evaluation and Research laboratory of the Food and Drug Administration USA, Human anti-yellow fever virus standard serum was obtained from Sanofi, sheep anti-influenza virus (California strain) serum and individual monovalent human anti-dengue sera (I, II, III and IV) were purchased from Medicines and Healthcare Products Regulatory Agency (MHRA/NIBSC), United Kingdom (UK). Polioviruses (PV), Yellow Fever virus (YF) and Dengue virus (DV) were grown in-house. California H1N1, New Caledonia H1N1 and Wisconsin H3N2 viruses were obtained from CDC-IRR (Center of Disease Control Influenza Research Resources, Georgia USA). Primary anti-virus labeling antibodies against PV (mouse monoclonal #ab47802, ab47820 and ab47803) and YF (mouse monoclonal #ab22839) were purchased from Abcam Inc, USA. California H1N1 primary labeling antibody (mouse monoclonal #430141) was purchased from MyBiosource, anti-influenza A H1N1 specific antibody (Goat biotin conjugated #1307) and anti-influenza A H3N2 specific antibody (Goat biotin conjugated #1317) from ViroStat and DV primary labeling antibody (mouse monoclonal) was purchased from Biotem for each individual dengue serotype. Secondary biotinylated anti-species (anti-mouse IgG (H+L)) antibodies were obtained from Jackson Immunoresearch, USA. Premium grade streptavidin-phycoerythrin (SA-PE) was purchased from Leinco Technologies Inc, USA. Trypan Blue was purchased from Sigma-Aldrich, USA. All the sera samples were heat inactivated at 56°C for 2 hours prior to their use. Turkey whole blood in citrate buffer (Turkey with Alsevers as added anticoagulant, #7209403) was purchased from Lampire Biologicals Laboratories, PA, USA.

### Vero cell culture

Vero cells (CCL-81) were obtained from ATCC, USA. Cells were grown in Iscove’s Modified Dulbecco’s Medium (IMDM, ATCC) containing 4mM L-glutamine, 4500mg/L glucose and 1500mg/L sodium bicarbonate with fetal bovine serum (10%) and 1% penicillin-streptomycin-glutamine (Life Technologies Inc. USA). Cells were maintained at 37°C, 5% CO_2_ in a humidified incubator. For fADI experiment cells were used at concentration of 1 million/ml at 50μl/well.

### fADI assay on Bioplex with standard animal and human serum

fADI was carried out in clear 96-well round bottom plates (Corning Inc., USA). All the sera dilutions were made in 1%BSA (Bovine serum albumin, Sigma-Aldrich USA) as well as all the primary antibody, secondary detecting antibody, SA-PE and Trypan blue solution were made in 1%BSA. In the first step, 25μl of serially diluted standard sera starting at 1:125 to 1:1000 (I, II and III) was incubated with corresponding 25μl of 2μg/ml of PV (I, II and III) in duplicate wells for 1 hour at 4°C on a shaker at 600rpm. After 1 hour, 50μl/well of Vero cells (1million/ml in 1%BSA) were introduced and mixed gently with pipetting and incubated for 1 hour at 4°C. This gives the final virus concentration of 0.5μg/ml and final sera dilutions of 1:500 to 1:4000. After 1 hour, cells were centrifuged at 600g for 4 minutes. Then supernatant fluid was aspirated and pellet was washed with 100μl/well of PBS/NaN3. Again plate was spin down at 600g for 4 minutes. Then supernatant was aspirated and cells were re-suspended in 100μl/well of 5μg/well of virus-specific mouse monoclonal antibodies, anti-PV I, anti-PV II and anti-PV III respectively in wells having PV I, II and III standard sera and incubated on shaker at 4°C at 600rpm. After 30 minutes, cells were washed with PBS/NaN_3_ as in previous steps. Then supernatant was aspirated and cells were re-suspended in 100μl/well of 5μg/ml of biotinylated-anti-mouse detecting antibody and incubated on shaker at 4°C for 30 minutes at 600rpm. Then similar spinning and washing procedure as discussed earlier was performed and cells were suspended in 100μl/well of SA-PE (10μg/well) covering plate with black cover. After incubation for 30 minutes, spinning and washing was performed with PBS/NaN3. Cells were re-suspended and washed with 100μl/well of 0.15%v/v Trypan Blue in 1%BSA. Finally, cells were re-suspended in 100μl/well of Trypan Blue solution and read on Bioplex used in this case as a micro-Facs device. Mild staining of the target cells by low concentration of Trypan Blue is needed to make the cells readable by the BioPlex, thanks to weak red and infrared fluorescence of Trypan Blue. Without this staining, the cells would be considered by BioPlex as contaminating objects not fitting the fluorescence regions designated for Luminex beads. In effect, using such staining the cells are disguised for Luminex beads.

Identical procedure was applied for YF and California H1N1 standard serum except different mouse monoclonal, anti-yellow fever and anti-California H1N1; primary-labeling antibodies were used respectively against YF and California virus at concentration of 5μg/ml, 100μl/well. YF and California H1N1 viruses were used at final concentration of 5μg/ml and 2μg/ml respectively while final sera dilutions ranges were 500 to 4000 and 1000 to 8000 respectively. DV was used at final concentration of 10μg/ml and strain-specific mouse monoclonal were used against each DV (I, II, III and IV) as primary antibody at 5μg/ml, 100μl/well while final dilution range for each monovalent serum was 50 to 2700. Rest of the procedure remains same as described above.

### ELISA with standard animal and human sera

The control ELISA was carried out using exactly same experimental conditions as described for the main Bioplex fADI assay except virus was coated on Immulon strips instead of using Vero cells. The detailed procedure is as descried here; ELISA was carried out by coating the Immulon 4HBX strips with 100μl/well of 2μg/ml PV (I, II and III) solution in PBS/NaN3 in duplicates, overnight at 4°C. After 12hrs the plates were blocked with 200μl/well of 2%BSA for 2 hours. Then the plates were washed twice with PBST (Phosphate Buffer Saline with 0.05% Tween 20) and serially diluted standard sera were introduced respectively. After incubation for 2 hours, the plates were washed with PBST twice, and mouse monoclonal anti-polio virus antibody was added at 5μg/ml, 100μl/well, and the plates were incubated for 1 hour at 4°C. Next plates were washed twice with PBST and anti-mouse IgG(H+L)Fab2:biotin antibody was added at 5μg/ml, 100μl/well, and the plates incubated for 1 hour at 4°C. Next, the plates were washed with PBST thrice and incubated for 1 hour at 4°C with 50μl/well of SA-PE at 10μg/ml. Control wells did not contain any sera (no sera) and blank wells were not coated with virus. Finally, the plates were washed with PBST four times and read on BioTek plate reader using phycoerythrin filter setting.

Identical procedure was applied for YF and California H1N1 standard serum except different mouse monoclonal, anti-yellow fever and anti-California H1N1; primary-labeling antibodies were used respectively against YF and California virus at concentration of 5μg/ml, 100μl/well. YF and California H1N1 were used at final concentration of 5μg/ml and 2μg/ml respectively while final sera dilutions ranges were 500 to 4000 and 1000 to 8000 respectively. DV was used at final concentration of 10μg/ml and strain-specific mouse monoclonal were used against each DV (I, II, III and IV) as primary antibody at 5μg/ml, 100μl/well while final dilution range for each monovalent serum was 50 to 2700. Rest of the procedure remains same as described above. All the experiments were carried out in duplicates.

### Classical HAI

In a typical 96-well flat-bottom polyprostyrene microplate 50μl of PBS/NaN3 was added to all wells except column 1. Then 100μl of pre-diluted sera samples (pre- and post-immunized sera starting dilution of 1:20) or standard were added in the column 1. Then sera/standard were diluted vertically in two-fold serial dilutions (starting dilution for sera was 1:20), discarding the last 50μl portion taken from last sample and standard wells. Control wells contain no serum and no virus. Next, 50μl of pre-diluted virus (1:20) was added to all wells except to no virus wells (negative control), as determined by Classical HA and 50μl of PBS/NaN3 added to no virus wells. Afterwards plate was placed in a planar plate shaker and mixed for approximately 5 seconds at 1000 rpm. It was repeated for a total of three times. Then plates were covered and incubated for 1 hour at 500 rpm at 4°C. After 1 hour, 50μl of processed erythrocytes diluted at 1.5% HCT (1:67) in PBS/NaN3 added to all wells. Then the sample plates were mixed for approximately 5 seconds at 1000 rpm. This procedure was repeated for a total of three times. Finally, plates were left undisturbed on the bench 30 to 60 minutes to allow erythrocytes to precipitate and form the hemagglutination patterns, reading the plate(s) with the Autoimmun Diagnostika GmbH (AID, Germany) plate reader.

### Classical HAI with New Caledonia H1N1 and Wisconsin H3N2 influenza viruses for human donor immunized with 2006–2007 seasonal Influenza vaccine

Human pre- and post-vaccinated sera were collected from donors vaccinated against seasonal influenza vaccine 2006–2007. A total number of 18 human donor sera (36 pre- and–post) were screened for both strains of virus using fADI and HAI. Both viruses were used at final concentration/dilutions of 1 to 3200 and 1 to 400 for New Caledonia H1N1 and Wisconsin H3N2 respectively. Sera were tested over a range of titrations ranging from of 1:3200 to 1:28800. The final procedure was conducted as described in the above section, except goat biotinylated-anti-influenza A H1N1 and biotinylated-anti-influenza A H3N2 antibody were used to label virus and processed turkey erythrocytes were used as target instead of Vero cells. fADI 50% titers were determined using the titration curve. Control wells contained no sera and blank wells had neither sera or virus. A classical HAI assay was performed for both virus strains using the same sera samples as described in above section except Human erythrocytes (Group O) were used instead of turkey erythrocytes.

### Classical Hemagglutination Inhibition Assay (HAI) for California H1N1. Classical Hemagglutination (HA)

Same donor sera vaccinated with influenza vaccine as screened for fADI and control ELISA were used for classical HAI assay. Prior to HAI, viral HA titer for California H1N1 was determined as described here;

In a typical 96-well flat-bottom polyprostyrene microplate 50μl of PBS/NaN3 was added to all wells except column 1. Then 100μl of pre-diluted (1:10) California H1N1 virus was added to the wells of column 1 in duplicate. Then virus was diluted in two-fold serial dilutions, discarding the last 50μl portion taken from last sample and standard wells. Control wells contained no virus. Then 50μl of PBS/NaN3 was added to all wells. Next, plate was placed in a planar plate shaker and mixed for approximately 5 seconds at 1000 rpm. It was repeated for a total of three times. Afterwards, 50μl of processed erythrocytes diluted at 1.5% hematocrit (HCT) (1:67) in PBS/NaN3 was added to all wells (Processing of erythrocytes; a fresh samples of turkey blood balanced with citrate buffer were washed three times in PBS (at 400 g), and the upper layer of the pellet containing lymphocytes was discarded. The washed erythrocytes were diluted in 1% human serum albumin (HSA) in PBS to the level of 0.03% HCT). Then plate was mixed by placing in a planar plate shaker and mix for approximately 5 seconds at 1000rpm. It was repeated for a total of three times. Subsequently, plate was left undisturbed on the bench for 30 to 60 minutes to allow erythrocytes to precipitate and form the hemagglutination patterns. Finally, the plate was read on the AID plate reader. Determined viral HA titer was subsequently used for classical HAI.

### Poliovirus fADI and Control ELISA for human clinical serum vaccinated with IPOL^®^ in 2009

fADI for PV (serotypes I, II and III) human clinical sera was performed using exactly the same experimental conditions as described in the previous section of standard serum fADI. Human sera from individuals before and after vaccination with IPOL^®^ were tested at a final dilution of 1:1000 with 0.5μg/ml each virus serotypes (PV I, II and III) individually. Total number 60 sera samples (pre and post) from 30 human donors were tested in duplicates. fADI 50% titer was calculated using the mathematical model as described. Similarly, the control ELISA was performed using exactly same condition as reported in earlier section, coating the Immunlon strips with 2μg/ml, 100μl/well, of all three serotypes individually. Subsequent steps and procedure were followed as described in above section of standard serum control ELISA.

### Polio microneutralization assay

Microneutralization was performed at CBER/FDA. Same sera samples as used in PV fADI were subjected to MN assay using Vero cells as target. MN assay was carried out using the procedure as outlined in the World Health Organization (WHO) Manual for the virological investigation of polio under section 6.3 entitled as neutralization test for polio antibodies and MN 50% titers were calculated as described in the manual. All sera samples were frozen and transferred to the CBER laboratory.

### fADI and control ELISA with YF-VAX^®^ vaccinated human sera samples

Human pre- and post-vaccinated sera were collected from donors vaccinated against yellow fever with YF-VAX^®^ 2009 vaccine. A total number of 24 human donor sera were screened for both fADI and control ELISA. Yellow Fever virus was used at final concentration of 5ug/ml and sera were tested at final dilution of 1:500. Remaining procedure was followed as described in the above section of fADI for polio, except using mouse monoclonal anti-yellow fever antibody at 5μg/ml, 100μl/well instead of anti-polio antibody. fADI 50% titers were determined using the mathematical model.

Control ELISA was performed using exactly same ELISA procedure as described in above section by coating the Immulon strips with yellow fever virus at concentration of 5μg/ml in PBS/NaN_3_, 100μl/well. Control wells contain no sera samples and blank wells were not coated with virus.

### PRNT50 for YF-VAX^®^ vaccinated human sera samples at Focus Diagnostics Inc, California, USA

Pre- and post-vaccination sera collected from YF-VAX vaccinated individuals (as used in YF fADI) were used to determine the PRNT50 titer. PRNT was performed independently by outside commercial vendor Focus Diagnostics, using the proprietary procedure in their laboratory in California. All sera samples were frozen and transferred to Focus Diagnostics. PRNT 50% titers were determined by applying the Linear Regression analysis.

### California H1N1 fADI and Control ELISA for human donor immunized with 2013–2014 seasonal influenza vaccine

Human pre- and post-vaccination sera were collected from individuals vaccinated against seasonal influenza in 2013 and against pandemic California H1N1 virus. A total number of 28 human donor sera were screened for both fADI and control ELISA. California H1N1 virus was used at a final concentration of 2μg/ml and sera were tested at final dilution of 1:1000. The final procedure was followed as described in the above section, except anti-California H1N1 mouse monoclonal antibody was used as primary labeling antibody at 5μg/ml, 100μl/well. fADI 50% titers were determined using the VAI numbers and their conversion to titers via mathematical model. The control ELISA was performed using exactly same ELISA procedure as described in above section by coating the Immulon strips with California H1N1 virus at concentration of 2μg/ml, 100μl/well, in PBS/NaN_3_. Control wells contained no test sera and blank wells were not coated with virus.

### Automated and manual fADI

For correlation study between automated and manual fADI, 20 human donor sera immunized with IPOL^®^ 2009 vaccine were randomly selected and ran simultaneously in manual and automated mode (these 20 human sera were different than 30 donor sera used in earlier study).

The automated fADI assay was performed on a Beckman Coulter Biomek® Assay Workstation comprising a Biomek® FX^P^ liquid handler, Cytomat^TM^ Hotel for ambient microplate storage, Cytomat^TM^ 2 C470 microplate incubator at 4°C, BioTek ELx405^TM^ UCW microplate washer, 1m ORCA® robotic arm and a Hettich® Rotanta 46 RSC centrifuge. The method was developed using SAMI® EX scheduling software and custom scripting through Biomek® software. The automated fADI method begins with the addition of the Vero cells. Manually prepared assay plates containing serially diluted sera and virus from the first step discussed above were loaded onto the assay workstation following the initial 1 hour incubation, along with pipette tips and reservoirs containing assay reagents. The automated method then performed the remaining assay steps without manual intervention as follows. First, 50μl/well of Vero cells (1 million/ml) were re-suspended and transferred to the assay plates using the Biomek FX^P^ 96-channel pod. The 96-channel pod then mixed each well to ensure a uniform cell suspension. Generally, the cell suspension is maintained during the manual process by shaking during incubations. However, shaking was not possible with the Cytomat incubator, so an additional mix step using an INHECO® Thermoshake was added for the 1 hour incubation following the addition of the Vero cells, i.e. the assay plates were incubated for 30 minutes at 4°C in the Cytomat, mixed using the 96-channel pod and then incubated for an additional 30 minutes at 4°C. The assay plates were then washed by centrifugation at 1680 rpm (600g) for 4 minutes at 4°C in the Rotanta centrifuge followed by vacuum aspiration of the liquid from the assay plate and the addition of 100μl/well of PBS/NaN_3_ using the ELx405 microplate washer. The cells were then re-suspended using the 96-channel pod and the centrifugation and vacuum aspiration steps were repeated. The 96-channel pod then transferred 100μl/well of 5μg/ml virus-specific mouse monoclonal antibodies, anti-PV I, anti-PV II and/or anti-PV III and mixed to re-suspend the cells. After 30 minutes at 4°C in the Cytomat incubator, the PBS/NaN_3_ centrifugation wash step was repeated and the cells were re-suspended in 100μl/well of 5μg/ml biotinylated-anti-mouse detecting antibody using the 96-channel pod and incubated in the Cytomat at 4°C for 30 minutes. The PBS/NaN_3_ centrifugation wash step was repeated again and the cells were re-suspended in 100μl/well of SA-PE (10μg/ml) using the 96-channel pod and incubated in the Cytomat at 4°C for 30 minutes. The centrifugation wash step was then performed with 100μl/well of 0.15%v/v Trypan Blue in 1% BSA instead of PBS/NaN_3_ and the cells were re-suspended in 100μl/well of the Trypan Blue solution. Analysis of the fADI samples was performed offline using the same Bio-Rad Bio-Plex® 100 multiplex array reader in MicroFacs mode as was used for the manually prepared samples.

## Supporting Information

S1 Fig Comparing fADI for NHP sera challenged with filoviruses and sera from human recipients immunized with influenza. Panel a: fADI for NHP sera challenged with Ebola Zaire (blue bars–pre-challenged serum; red bar–post-challenge serum, Marburg Musoke (green bars—pre-challenged serum; purple bars–post-challenge serum). Panel b: fADI for sera from human recipients immunized with seasonal influenza vaccine, 2009. Blue bars–pre-vaccinated serum, red bars–post-vaccinated serum. Strong pre-attachment blocking for influenza and lack of pre-attachment blocking for filoviruses are obvious.
(TIF)

S2 Fig Comparing fADI and HAI data for sera from human recipients immunized with seasonal influenza vaccine in 2009. Panel **a:** fADI and HAI data for New Caledonia H1N1 virus. Panel **b:** fADI and HAI data for Wisconsin H3N2 virus. Both fADI and HAI data shown as 50% titer values.
(TIF)

S3 Fig Theoretical fADI titration curves for strong and weak sera. **Serum A** is supposedly weak. **Serum B** is supposedly strong. For both weak and strong sera, the ***shapes*** of the titration curves are supposed to be similar. **Red curve** shows the titration curve symmetrized mathematically to center at Log(dilution) = 0 (i.e., a serum is undiluted).
(TIF)

S4 Fig Stepwise determination of the symmetrized titration curve for polio fADI. **a:** Step 1, plotting a few primary VAI data against dilution factor; **b;** Step 2, re-plotting the primary VAI data in semi-Log coordinates; **c:** Step 3, adding VAI data from other measurements, re-plotting; **d:** Step 4, fitting a sigmoidal regression curve to the plotted VAI data using Least Square Fitting method; **e:** Step 5, symmetrization fo the curve-fitted regression and the experimental data, i.e. finding the shift value that makes the center of the curve at 0 using Least Square Fitting method; **f:** dual scattering re-plotting of the symmetrized regression VAI points versus symmetrized experimental VAI data.
(TIF)

S5 Fig Transfer of the single point VAI data into fADI 50% titers using the symmetrized titration curve. **a:** VAI data determined for Polio 1 at dilutions 1:250 and 1:1000. **b;** fADI 50% titers calculated from the VAI data.
(TIF)

S6 Fig Comparing fADI 50% titers calculated from single point VAI data for Polio I, II and III viruses with the MN titers determined independently in CBER. **a, b, c:** fADI 50% titers calculated from the single-point VAI data at dilution 1:1000 for Polio I, II and III at dilution 1:1000. **d, e, f;** MN 50% titers determined independently in the FDA CBER.
(TIF)

S7 Fig Comparing fADI VAI and fADI 50% titers determined in the manual and automated fADI experiments. **a, b, c:** VAI data determined at dilution 1:1000. **d, e, f;** fADI 50% titers calculated from the VAI data.
(TIF)
