# Supplementary figures and images for: Fluorescence Adherence Inhibition Assay: A Novel Functional Assessment of Blocking Virus Attachment by Vaccine-Induced Antibodies
Source: PLoS One. 2016 Feb 10;11(2):e0144261. doi: 10.1371/journal.pone.0144261 (PMC4749260; doi:10.1371/journal.pone.0144261)

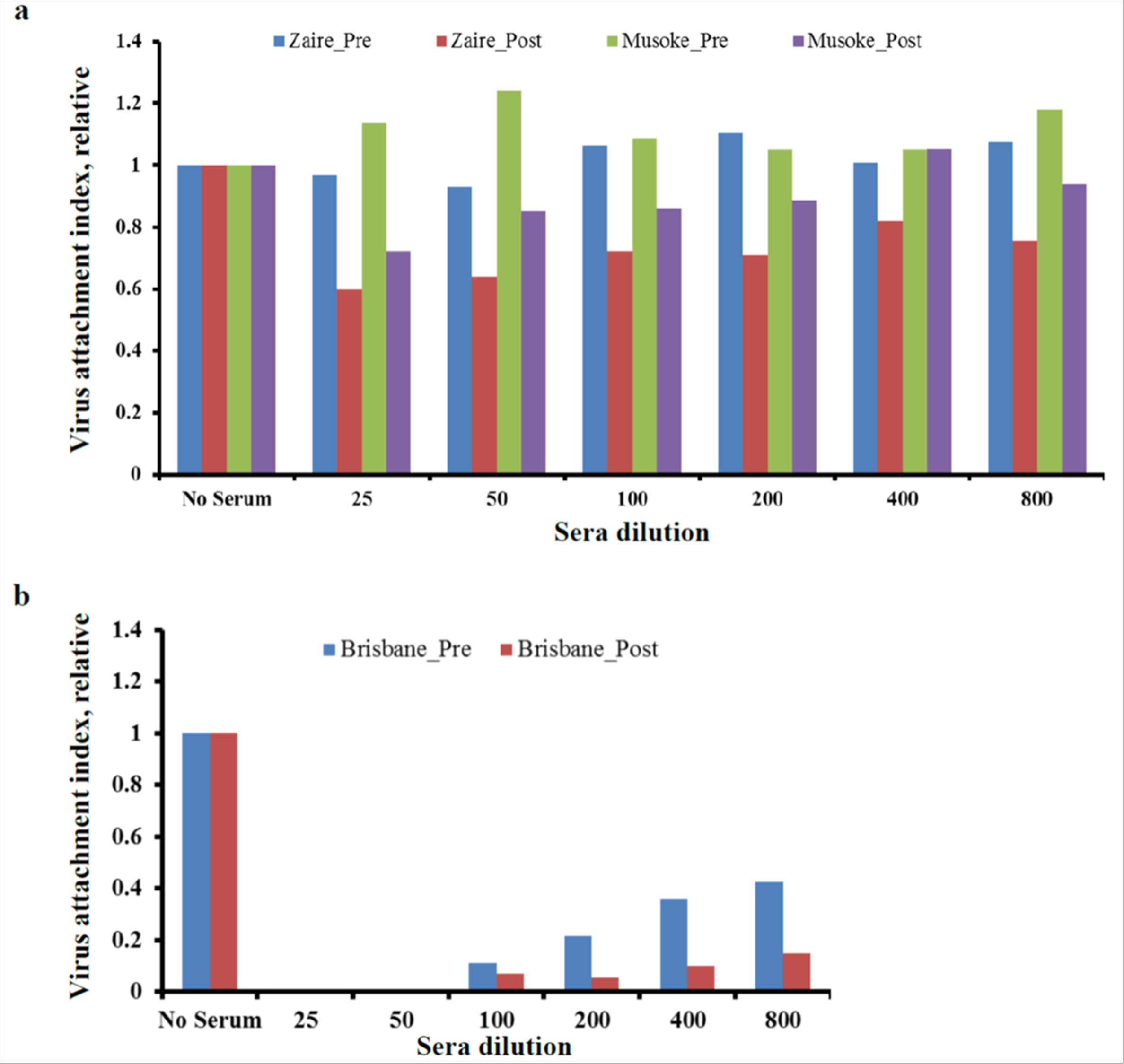

Supplement: S1 Fig — Panel a: fADI for NHP sera challenged with Ebola Zaire (blue bars–pre-challenged serum; red bar–post-challenge serum, Marburg Musoke (green bars—pre-challenged serum; purple bars–post-challenge serum). Panel b: fADI for sera from human recipients immunized with seasonal influenza vaccine, 2009. Blue bars–pre-vaccinated serum, red bars–post-vaccinated serum. Strong pre-attachment blocking for influenza and lack of pre-attachment blocking for filoviruses are obvious. (TIF) [file pone.0144261.s001.tif]

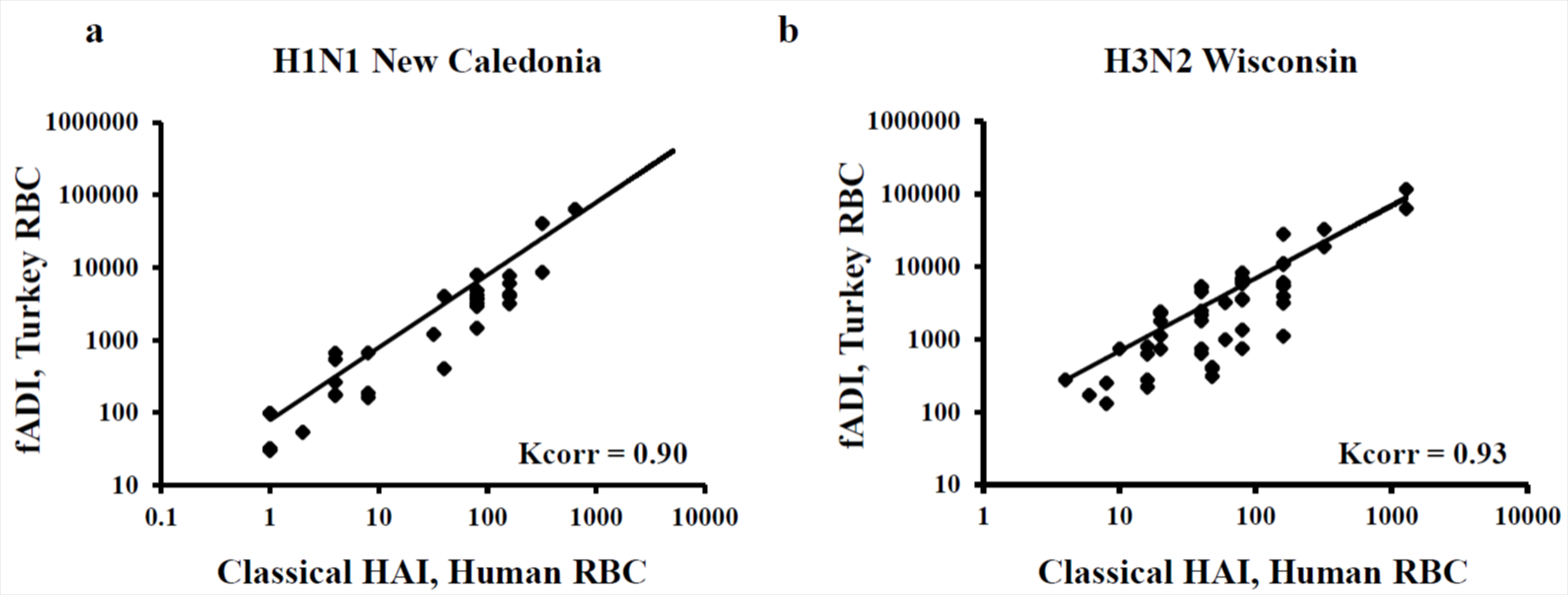

Supplement: S2 Fig — Panel a: fADI and HAI data for New Caledonia H1N1 virus. Panel b: fADI and HAI data for Wisconsin H3N2 virus. Both fADI and HAI data shown as 50% titer values. (TIF) [file pone.0144261.s002.tif]

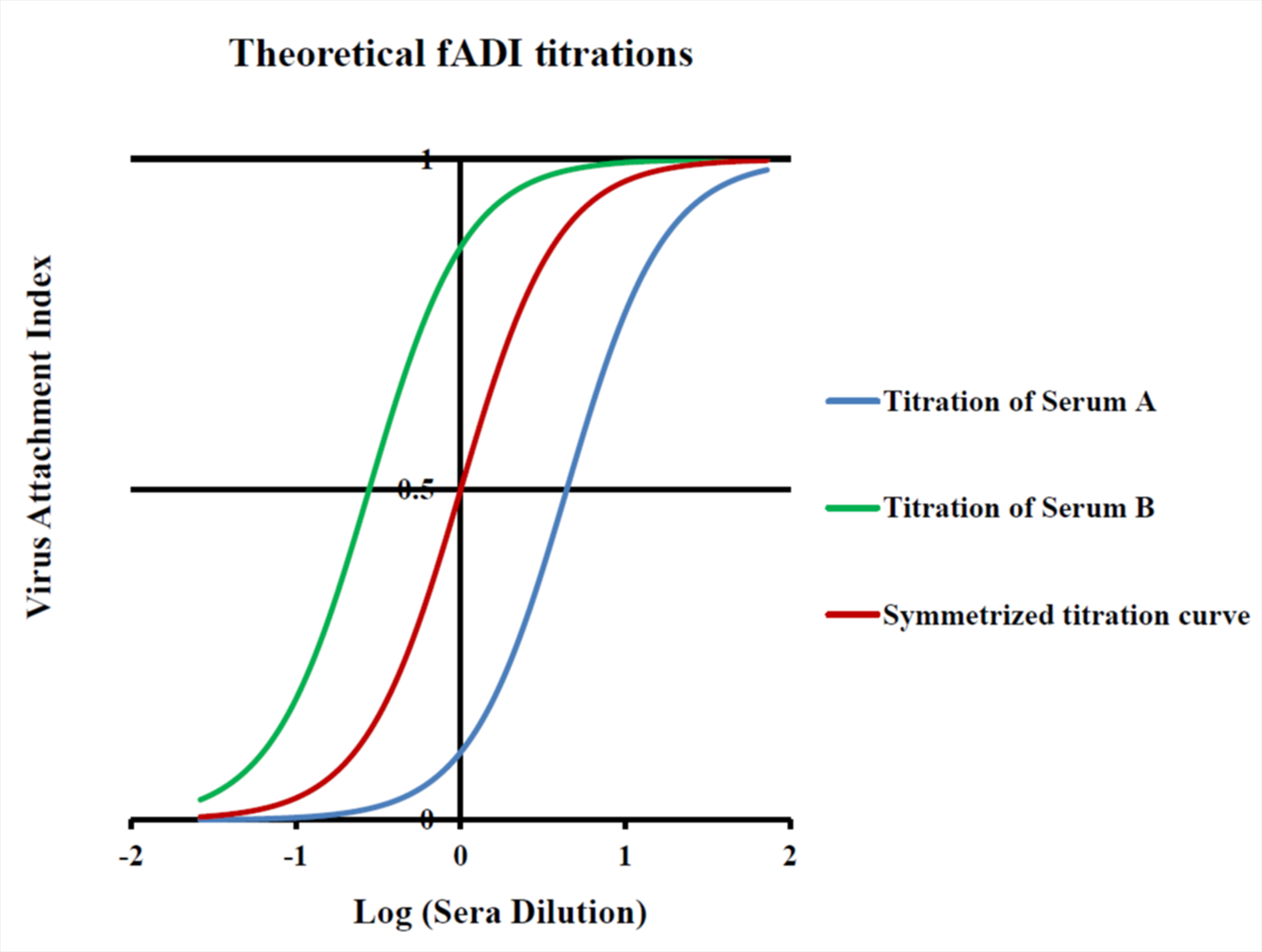

Supplement: S3 Fig — Serum A is supposedly weak. Serum B is supposedly strong. For both weak and strong sera, the shapes of the titration curves are supposed to be similar. Red curve shows the titration curve symmetrized mathematically to center at Log(dilution) = 0 (i.e., a serum is undiluted). (TIF) [file pone.0144261.s003.tif]

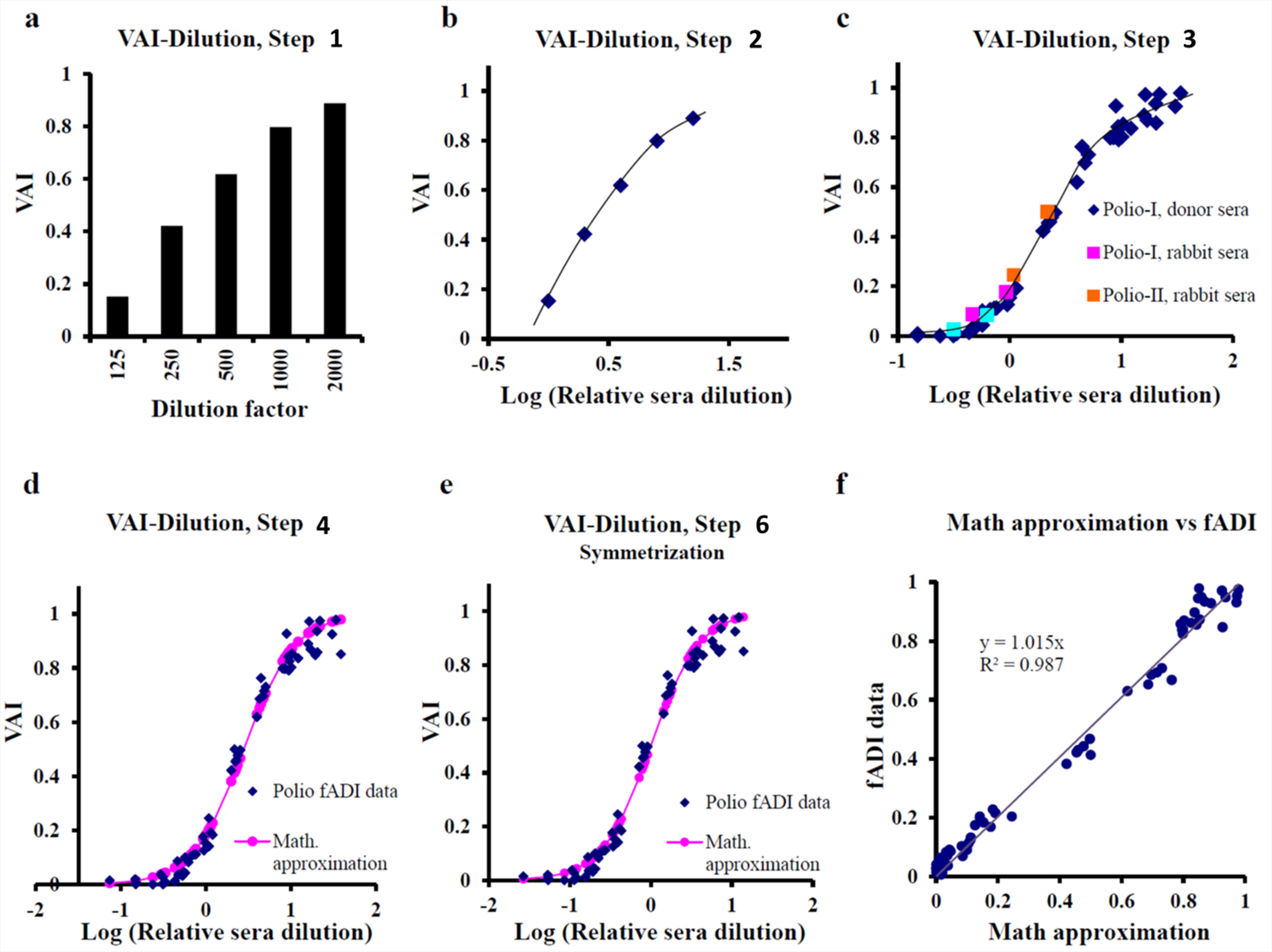

Supplement: S4 Fig — a: Step 1, plotting a few primary VAI data against dilution factor; b; Step 2, re-plotting the primary VAI data in semi-Log coordinates; c: Step 3, adding VAI data from other measurements, re-plotting; d: Step 4, fitting a sigmoidal regression curve to the plotted VAI data using Least Square Fitting method; e: Step 5, symmetrization fo the curve-fitted regression and the experimental data, i.e. finding the shift value that makes the center of the curve at 0 using Least Square Fitting method; f: dual scattering re-plotting of the symmetrized regression VAI points versus symmetrized experimental VAI data. (TIF) [file pone.0144261.s004.tif]

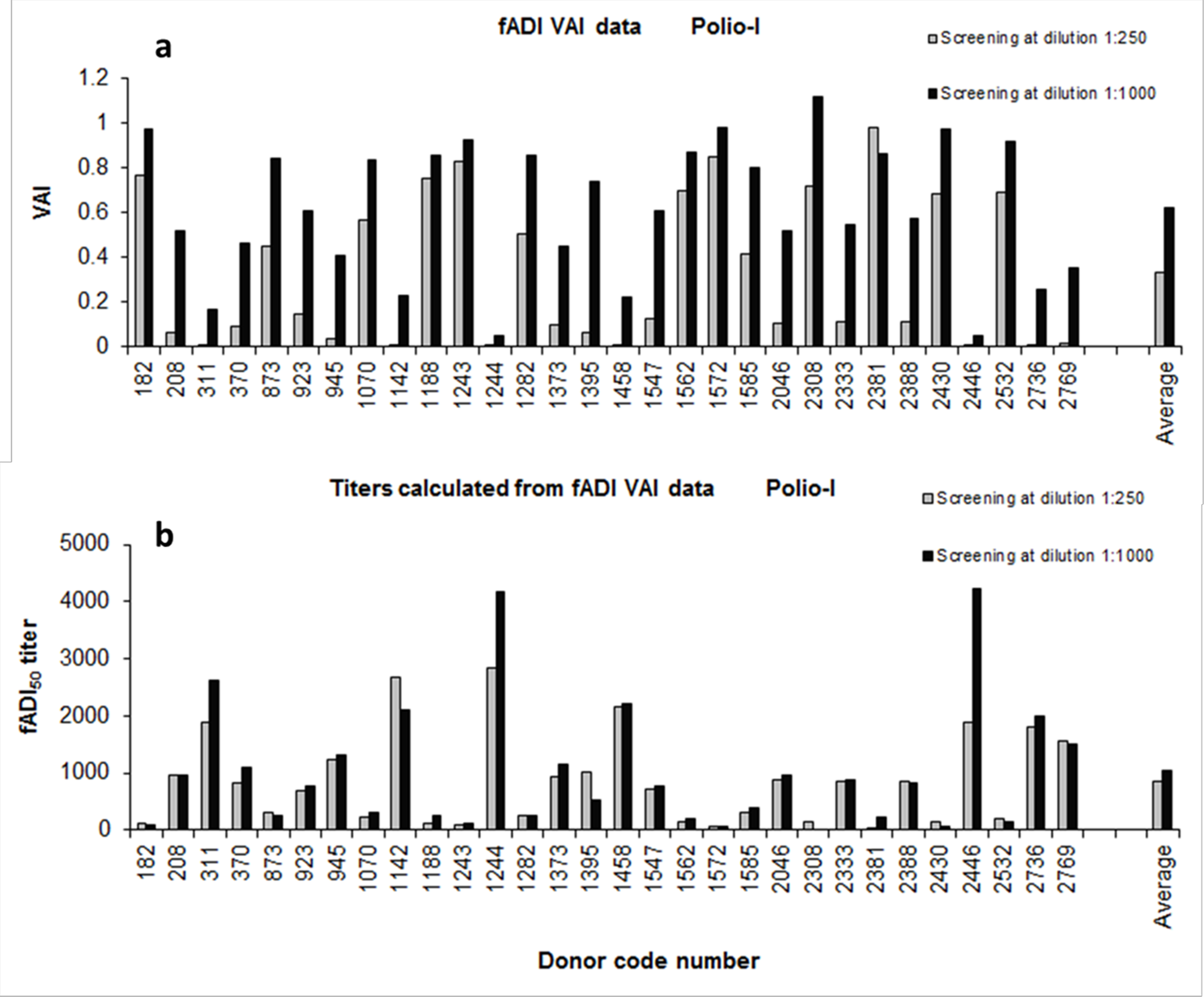

Supplement: S5 Fig — a: VAI data determined for Polio 1 at dilutions 1:250 and 1:1000. b; fADI 50% titers calculated from the VAI data. (TIF) [file pone.0144261.s005.tif]

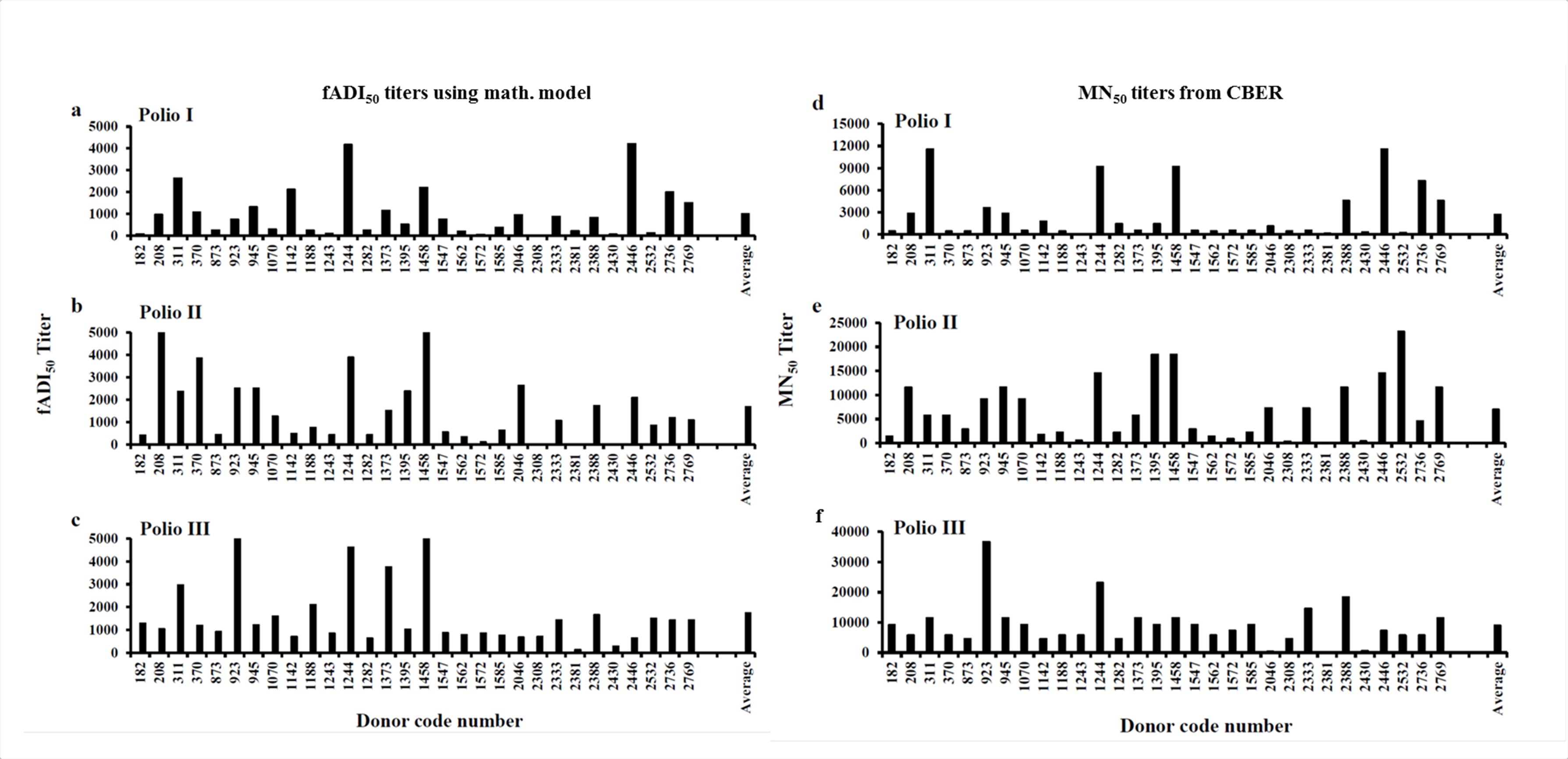

Supplement: S6 Fig — a, b, c: fADI 50% titers calculated from the single-point VAI data at dilution 1:1000 for Polio I, II and III at dilution 1:1000. d, e, f; MN 50% titers determined independently in the FDA CBER. (TIF) [file pone.0144261.s006.tif]

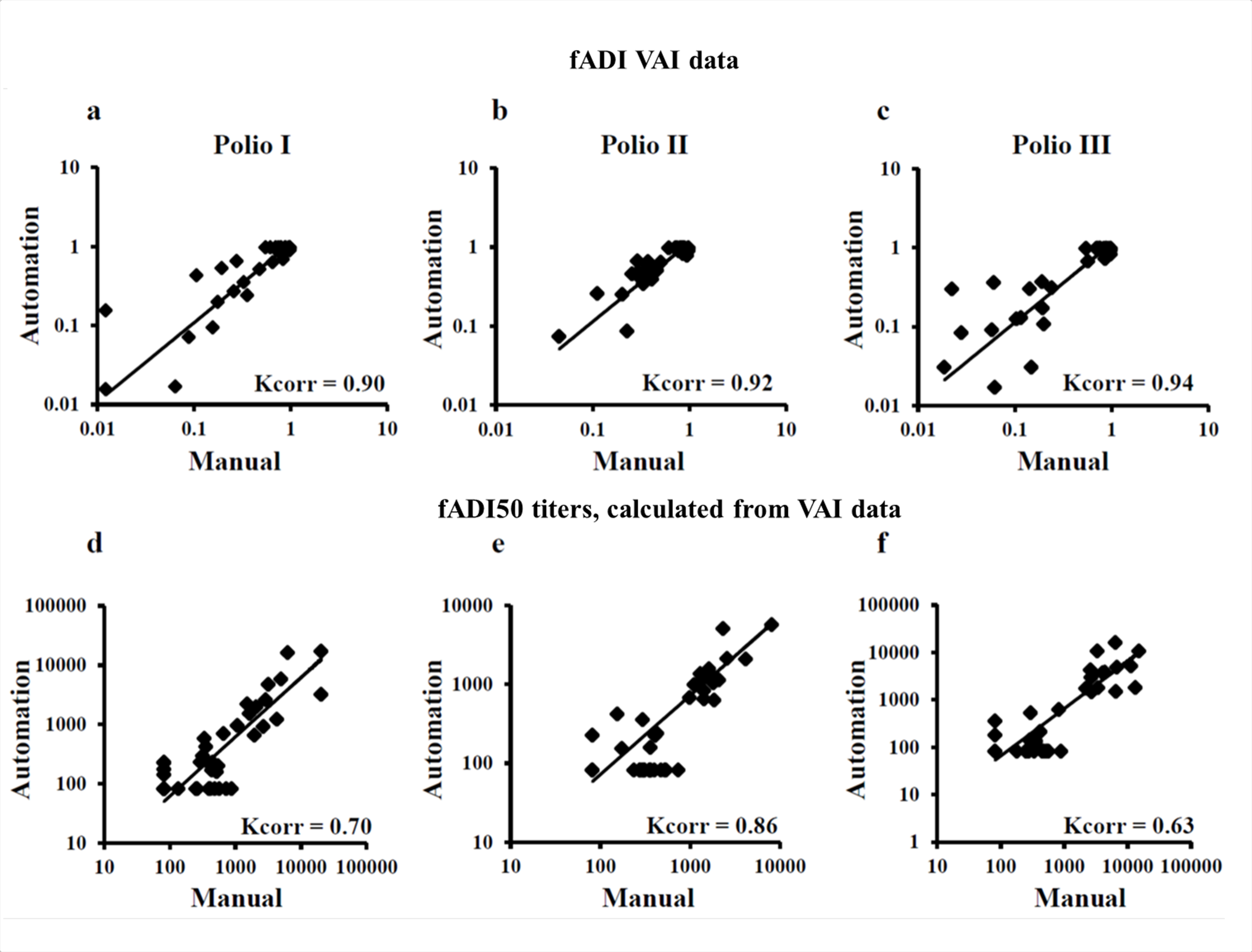

Supplement: S7 Fig — a, b, c: VAI data determined at dilution 1:1000. d, e, f; fADI 50% titers calculated from the VAI data. (TIF) [file pone.0144261.s007.tif]
